# “This Graft-vs.-Host Disease Determines My Life. That's It.”—A Qualitative Analysis of the Experiences and Needs of Allogenic Hematopoietic Stem Cells Transplantation Survivors in Germany

**DOI:** 10.3389/fpubh.2021.687675

**Published:** 2021-07-01

**Authors:** Mira Parisek, Julika Loss, Ernst Holler, Anna Barata, Daniela Weber, Matthias Edinger, Daniel Wolff, Helene Schoemans, Anne Herrmann

**Affiliations:** ^1^Faculty of Medicine, University of Regensburg, Regensburg, Germany; ^2^Department for Health Behaviour, Robert Koch Institute, Berlin, Germany; ^3^Department of Hematology and Internal Oncology, University Hospital Regensburg, Regensburg, Germany; ^4^Department of Hematology, Hospital de la Santa Creu i Sant Pau, Barcelona, Spain; ^5^Josep Carreras Leukemia Research Institute, Barcelona, Spain; ^6^Department of Health Outcomes and Behavior, Tampa, FL, United States; ^7^Department of Hematology, Universitaire Ziekenhuizen Leuven, Katholieke Universiteit Leuven, Leuven, Belgium; ^8^Department of Public Health and Primary Care, Katholieke Universiteit Leuven, Leuven, Belgium; ^9^Department for Epidemiology and Preventive Medicine, Division of Medical Sociology, University of Regensburg, Regensburg, Germany; ^10^School of Medicine and Public Health, University of Newcastle, Newcastle, NSW, Australia

**Keywords:** allogeneic hematopoietic stem cells transplantation, survivorship, patients view, framework analysis, qualitative research, GvHD

## Abstract

**Background:** Allogeneic hematopoietic stem cell transplantation (alloHSCT) is the only curative treatment modality for many patients affected by hematologic malignancies. However, it can cause debilitating long-term effects. Understanding the impact of alloHSCT on all aspects of the patients' life is required for optimal survivorship management.

**Aim:** To explore in-depth HSCT-survivors' experiences and needs post-transplant. Partners were included to provide further information on survivors' needs and how care could be improved in this area.

**Methods:** We conducted semi-structured face-to-face and phone interviews with alloHSCT-survivors and their partners referred to a survivorship clinic in Germany. Theoretical sampling was used to recruit participants. Data were analyzed using framework analysis.

**Results:** Thirty-two survivors (consent rate: 100%, response rate: 100%) and eighteen partners (consent rate: 84%, response rate: 72%) participated. Survivors were aged between 25 and 68 years (Median: 48, IQR: 25.3) and partners were aged between 26 and 64 years (Median: 54, IQR: 16, SD: 12.8). The themes emerging from the data involved survivors' needs included (i) the diversity of long-term treatment side-effects; and (ii) time post discharge as a dynamic process with individual peaks of burden. Survivors and their partners also suggested strategies for mitigating these unmet needs, i.e., (iii) transparent communication and patient empowerment; and (iv) improvement in continuity of care system and help with claiming social benefits as cornerstones of optimal survivorship care.

**Conclusion:** To our knowledge, this is one of the first qualitative studies focused on the views of German alloHSCT-survivors on the long-term effects of alloHSCT and the first study integrating the view of their partners. Healthcare providers could better support survivors with managing their symptoms and adhering to their prescribed care by ensuring comprehensive, transparent communication that helps increase survivors' understanding and involvement in their care. Further efforts should be made to provide patient-centered, continuous survivorship care that involves additional support with navigating the healthcare and social service system. Intervention studies are required to test the effectiveness of the suggested strategies.

## Introduction

Allogeneic hematopoietic stem cell transplantation (alloHSCT) is the only curative treatment modality for many patients affected by a number of hematological disorders, both malignant and non-malignant, including high-risk leukemia, lymphoma, myelodysplastic syndrome and myeloproliferative disorders (1–3). Around 20,000 patients receive alloHSCT in Europe each year with approximately half of them developing some form of acute and/or chronic graft-vs.-host disease (GvHD) (4). In 2020, more than 4,000 patients were treated with alloHSCT in Germany, receiving care in one of more than 60 specialized treatment centers. The vast majority of these treatment centers are part of university hospitals providing tertiary healthcare to both inpatients and outpatients (4). AlloHSCT involves chemotherapy and/or radiation therapy (conditioning), followed by the infusion of hematopoietic stem cells. Various adverse effects may appear from conditioning and particularly from the transplantation itself, including transplant related organ dysfunction, secondary malignancy, and serious infections. Managing these multiple complications are quite challenging for both physicians and survivors (5–7). A recent review of observational and intervention research focused on the unmet needs among patients with hematologic cancers and concluded that HSCT is a risk factor for great unmet needs (8). Boyes et al. found that patients with hematologic cancers also experience higher levels of psychological morbidity including symptoms of anxiety and depression, compared with patients diagnosed with other common cancer types, such as breast or prostate cancer (9).

AlloHSCT can further cause a number of long-term effects on survivors, including chronic GvHD (cGvHD) which affects up to 50% of survivors (10). This immunological disorder can impact on almost every organ system leading to a variety of symptoms, such as skin sclerosis, dry eyes and inflammation of the oral mucosa. Long-term toxicity may also impair neurocognitive function, bone density among other symptoms and thereby limit physical functioning and quality of live (10, 11). Due to improvements in treatment modalities and decreasing mortality rates following HSCT the number of survivors is increasing (12–14). While many HSCT-survivors struggle with self-management and adherence to their prescribed care (15) little is known about survivors' perspective on how long-term effects of alloHSCT impact on their lives, and how healthcare providers could better help them manage their symptoms and care (16, 17). For example, Laidsaar-Powell et al. conducted a recent review on adult cancer survivors and found that compared to other common cancers with relatively high survival rates, such as breast or prostate cancer, there is considerably little evidence about the perceptions and experiences of survivors of hematological cancers, particularly with regard to the long-term effects of alloHSCT, such as cGvHD (18–20).

One of the crucial resources in coping with the long-term sequelae of transplantation are resorting to support from partners (21). Partners commonly help survivors recall information delivered by their treating clinicians, provide guidance on treatment decision making and support survivors with managing their care and doing daily activities post discharge (22). Partners can provide important additional information on survivors' needs and wishes as they are often the most important part of survivors' social network and one of their main sources of information, advice and support (22–24). Also, involving survivors' supportive others, are cornerstones of optimal patient-centered care which has become the gold standard of care delivery (25, 26). However, only few studies include data provided by partners, and to our knowledge, none has focused specifically on the long-term needs and wishes of alloHSCT-survivors and also included their partners' views in relationship to survivors' needs and care using a qualitative approach (27). Most studies in this area investigate specific aspects of survivors' and partners' needs such as QoL, focus on partners' own needs or use quantitative methods to explore survivors' and partners' views (28).

Research is needed that examines the dynamics and interrelation of survivors' and their partners' views, experiences and needs related to optimal survivorship care following alloHSCT (20). Qualitative research is particularly suited to fill this gap as it provides valuable in-depth insights into survivors' perceptions of their care and thus enhances our understanding of existing quantitative data on hematological cancer survivors' views (29). Conducting qualitative research can further help discover areas for improvement of care and inform the development of interventions to help survivors better cope with their symptoms (30, 31). Yet, only few qualitative studies explored alloHSCT-survivors' experiences post-transplant (19, 32–37), with many of them focusing on survivors of leukemia or lymphoma (33). Also, there is still a lack of research on how care in this area could be improved and, to our knowledge, very few qualitative studies have focused on the views and perceptions of European alloHSCT-survivors taking into account that findings from other contexts may not be generalizable due to differences in healthcare settings and services. We therefore performed qualitative semi-structured interviews with German alloHSCT-survivors and their partners to provide in-depth insights into survivors' views on how long-term effects of alloHSCT impact on their lives, and how healthcare providers could better help them manage their symptoms and care.

## Methods

### Aims

This study explored in-depth the alloHSCT-survivors' experiences and needs post-transplant. Partners were included to provide further information on survivors' needs and how care could be improved in this area.

### Study Design

Semi-structured face-to-face and phone interviews with alloHSCT-survivors and their partners were conducted.

### Participants

Survivors were eligible for this study if they (i) had received alloHSCT between 1 and 5 years prior to recruitment, (ii) were aged 18 years or older, (iii) were relapse-free after alloHSCT, (iv) were German speaking, (v) and visited the Dept. of Hematology of the University Hospital of Regensburg for routine follow-up visits or second opinion at the GVHD clinic. Survivors were able to participate regardless of their partner participation to ensure that the views of single survivors were also included in this study. The period of 1–5 years post alloHSCT was chosen as the first year after alloHSCT is often characterized by acute complications on treatment and thus not representative for long-term effects and outcomes of alloHSCT-survivors. The 5-year limit has been traditionally seen as a benchmark for cancer survivorship indicating a time where cancer recurrence becomes less likely (38–41). We only included relapse-free survivors as a relapse often leads to a disruption of care which is then not focused on survivorship but treatment of the underlying condition.

Partners were (i) identified by survivors as their partners, (ii) aged 18 years or older (iii) German speaking, and (iv) judged by clinic staff and the research team to be physically or mentally capable of completing the interview and signing the consent form.

### Recruitment

Participants were recruited between November 2019 and June 2020. The attending physician identified all eligible patients based on clinic records and asked for their consent to be contacted by a member of the research team to schedule the interview. Participants provided written informed consent by returning the signed consent form. Survivors' were asked whether they had a partner and if so, whether their partner would be willing to attend the interview. Survivors or partners were excluded after three unsuccessful attempts to contact them or if they declined to participate due to time constraints.

Theoretical sampling was conducted. Thus, initial consecutive data collection from clinic lists and analysis informed later recruitment to provide a maximum variation sample (42). Thus, survivors from various age and gender groups, as well as different socio-economic and disease-related backgrounds were recruited to collect heterogeneous cases in order to compare and progress the initial codes and categories (43). Ethical approval was granted by the local research ethics committee (approval no.: 19-1306-101).

### Data Collection

Participants could choose whether to conduct the interview face-to-face or via phone to reduce research-related burden on participants. Since the start of the COVID-19 pandemic, all participants have opted for phone interviews. Interviews were conducted by two interviewers (MP and AH), either one-on-one or with survivors and partners interviewed together. Consent was requested to record and transcribe the interviews. Participants were advised that they had the possibility to stop or to postpone the interview at any time. At the beginning of each interview participants were asked to talk about the impact the transplant had on their lives. This open stimulus helped elicit the myriad of factors associated with the time post-transplant. The narrative was followed by semi-structured questions examining topic areas not raised about by participants initially. Topic areas included physical and emotional well-being and needs, information provided to survivors along the care continuum, difficulties in daily routine and practical issues (such as keeping the household or baying bills), impact on relationships, and social and medical support provided by the healthcare team (for detailed information in each topic area please see the interview guide provided in Appendix 1). Partners were prompted to add to the survivor's narratives if they felt they would like to elaborate on certain aspects further. The interview guide was developed by the research team based on a review of the literature and further interdisciplinary discussion. It was also informed by the Supportive Care Framework (44). The framework was developed as a tool for cancer care professionals to help identify the type of support cancer survivors may require and how to meet these needs in healthcare service delivery (44). Employing a theoretical framework helped summarize and integrate existing knowledge on supportive care to provide guidance for research and clinical practice specific to the experiences and needs of alloHSCT-survivors (45).

### Data Analysis

All interviews were transcribed verbatim. Data were analyzed using the framework method which is a systematic approach for examining commonalities and differences in qualitative research data (46). It allows to provide highly structured outputs by summarizing descriptive and explanatory data and categorizing this data into developed topics (46). At the beginning of the analysis, the data were read intensively to identify generic terms that were clustered into categories (46). This step is called “open coding” and involves translated passages of the transcribed interview into key messages, which are then grouped into categories. The developed system of categories was discussed and double-checked within the research team. Once the system of categories (“the framework”) had been agreed on, the content of all interviews was scanned to assign further data to the framework (46). If additional topics repeatedly appeared, the analytical framework was constantly revised and completed until a final category-system was achieved (i.e., no additional new information arising). Again, the system of categories was discussed by the research team. This dynamic and iterative categorization allowed for the analysis of data in a detailed manner and enabled us to establish links between the categories. The final output of the analysis was summarized in the form of themes which were developed by interrogating the categories through comparison within and between interviews in order to form interpretive concepts that describe or explain certain aspects of the data (47).

## Results

Thirty-two survivors and twenty-five partners were approached (seven patients indicated that they were not in a relationship). All survivors agreed to participate, and eighteen partners contributed to the study (consent rate: 84%, response rate: 72%). Reasons for declining study participation were time constraints (*n* = 5) or lack of interest in this study (*n* = 2). Survivors were aged between 25 and 68 years (Median: 48; IQR: 25.3; SD: 14.1) and partners were aged between 26 and 64 years (Median: 54; IQR: 16). The median time since allogenic HSCT was 2.7 years (range: 1.2–5 years, IQR: 1.3, Tables 1, 2). Interviews lasted between 25 and 88 min (Median: 50.4, IQR: 25.2).

**Table 1:** AlloHSCT survivors' sociodemographic and disease related characteristics.

**Characteristics**	**Survivors**	
***N***	**32**	**In %**
**Gender**		
Female	16	50%
Male	16	50%
**Nationality**		
German	31	97%
Others	1	3%
Median Age in years (SD)	48.0	14.1
18–39 y	11	34%
40–59 y	11	34%
60+ y	10	31%
**Material status**		
Married	21	66%
Stable Relationship	4	13%
Single/widowed	7	22%
**Highest level of education**		
No university degree^*^	22	69%
University degree	10	31%
**Occupation**		
Full-time	6	19%
Part-time	9	28%
None/pension	15	47%
**Children**		
Non-Adult Children	12	38%
Adult Children	8	25%
None	12	38%
Median Time post HSCT in years (SD)	2.5	1.1
1–2 y	8	25%
2–3 y	15	47 %
3–4 y	3	9%
4–5 y	6	19%
**Diseases**		
Acute myeloid leukemia	9	28%
Acute lymphoblastic leukemia	7	22%
Non-Hodgkin Lymphoma	5	16%
Chronic myeloid leukemia	3	9%
Lymphoma	2	6%
Myeloproliferative disease	2	6%
Inborn immunodeficiency	2	6%
Others	2	6%
**cGvHD at time of interview (clinician reported)**		
None	10	31%
Mild	2	6%
Moderate	9	28%
Severe	11	34%

* *Year 10 or lower, A levels*.

**Table 2:** Partners' sociodemographic characteristics.

**Characteristics**	**Survivors**	
***N***	**18**	**In %**
**Gender**		
Female	12	67%
Male	6	33%
**Nationality**		
German	18	100%
Median Age in years (SD)	54.0	12.8
18–39 y	4	22%
40–59 y	9	50%
60+ y	4	22%
**Highest level of education**		
No university degree^*^	12	67%
University degree	6	33%
**Occupation**		
Full-time	8	44%
Part-time	5	28%
None/pension	5	28%
**Living with patient**		
Yes	18	100%

* *Year 10 or lower, A levels*.

The themes that emerged from the data of survivors and partners were grouped around needs, i.e., (i) the diversity of long-term treatment side-effects as a major challenge in alloHSCT survivorship care; (ii) time post discharge as a dynamic process with individual peaks of burden; and strategies for clinical practice to meet these needs, i.e., (iii) transparent communication and support with patient empowerment; and (iv) continuity in the care system and help with claiming social benefits as cornerstones of optimal survivorship care. These themes are described in detail below.

A summary of the themes and suggestions for how to improve care can be found in Table 3.

**Table 3:** Main themes and suggestions for clinical practice.

**Issues mentioned**	**Possible approaches**
Diversity of long-term treatment side-effects as a major challenge in alloHSCT survivorship care Time post discharge as a dynamic process with individual peaks of burden	1. Increased education of survivors and support persons about side-effects of alloHSCT prior to transplant and guidance on how to identify and communicate concerns and wishes 2. Continuing medical education on how to provide specialized care post-transplant 3. Provision of individually tailored survivorship care plans and easy access to multidisciplinary care teams
Transparent communication and support with patient empowerment	1. Healthcare provider training in patient-centered communication 2. Fostering patient self-management through involvement of liaison nurses 3. Provision of further publicly available information about treatment centers 4. Access to revalidation programs (e.g., as online course to reduce risk of infection)
Continuity in the care system and help with claiming social benefits as cornerstones of optimal survivorship care	1. Patient navigators and bridging nurses to coordinate continuous, individually tailored care, help navigate social support systems and maintain and strengthen communication and collaboration between all member of the multidisciplinary care teams

### Diversity of Long-Term Treatment Side-Effects as a Major Challenge in alloHSCT Survivorship Care

Survivors and partners reported great diversity of long-term side-effects of alloHSCT and varying degrees of burden caused by each side-effect of treatment. Given the complexity of GvHD they indicated that such care should ideally be provided by a clinic or center that is specialized in this area. Survivors attending specialized clinics felt that they received care that was more tailored to their individual needs and circumstances. The most frequently mentioned complication survivors struggled with was cutaneous cGvHD causing skin erythema, dryness often resulting in lasting visible transformations of different parts of the skin. A number of survivors reported having “typical GvHD spots” (female survivor, 57 years, two and a half years post alloHSCT) and described a great degree of suffering from a very dry and sometimes itchy feeling, getting worse during sun exposure. Some participants reported that the mucous membranes of the intimate area were affected by the GvHD causing pain during sexual intercourse.

“*It doesn't hurt, but it is so visible – skin looks like parchment and it dries out relatively quickly. [.] and then I have these spots. This was the first thing that we noticed.” (male survivor, 36 years, three years post alloHSCT)*

“*My mucous membrane was quite affected. It was as if my vagina was glued together.” (female survivor 47 years, four years post alloHSCT)*

Even though cutaneous cGvHD is a common problem, the perceived burden caused by this complication varied significantly between survivors. Women often reported being more concerned about visible transformations of their body than men and reported “not feeling attractive” anymore (female survivor, 46 years, 2 years post alloHSCT). Compared to male survivors, women also seemed to struggle more with the side-effects of long-term corticosteroids treatment, such as weight gain or facial oedema.

“*My skin is covered with brown spots. They are scars, they won't go away anymore. It is particularly bad when that happens on the neck and in the face. […] Especially for me as a woman it is not so easy to come to terms with that.” (female survivor, 57 years, two and a half years post alloHSCT)*

*You feel bad anyway as a cancer patient, without hair and so on. And after the transplant your whole appearance changed. And then you don't want to take cortisone in huge doses because you gain 20 kilos. […] It is the outward appearance especially for women. You would like to be like you were before [*=*alloHSCT]. (female survivor, 46 years, two years post alloHSCT)*

Even years after receiving alloHSCT, most survivors perceived a reduced level of general fitness and self-effectiveness compared to the time prior to the transplant. Some described this as a reduced capacity to deal with problems of daily life or to do daily activities, such as housekeeping or paying bills. Most reported lacking physical strength, suffering from chronic fatigue and reduced cognitive function. Thus, a significant part of survivors found it hard to concentrate on a task for a longer period of time. They also struggled with deciding what activities they are capable of doing and what would be too much for them. This often resulted in having to cancel appointments on a short notice and taking numerous breaks during the day. Partners emphasized this loss of fitness and highlighted survivors' need to take regular breaks, e.g., in the form of frequent and long naps during the day.

“*I feel a complete lack of energy. When I have to get up the stairs, I feel like I have been climbing Mount Everest.” (male survivor, 42 years, one and a half years post alloHSCT)*

“*For me the problem was exhaustion, tiredness. […] I was unsure how to handle that lack of energy and strength.” (female survivor, 51 years, two years post alloHSCT)*

“*I realize that my diary fills up quickly and then afterwards I have to cancel appointments because I simply don't have enough energy. And then I have to make sure that I don't have an appointment every day, let alone two in a day, that is much too overwhelming for me.” (female survivor, 57 years, two and a half years post alloHSCT)*

“*He sleeps a lot - a lot!” (female partner, 65 years)*

The majority of survivors who did not suffer from a significant change of their outer appearance noticed a discordance between how their health status was perceived by others and their own experience. This is due to the fact, that many symptoms they face, such as fatigue, reduced fitness-levels or mental stress were not visible from the outside. Survivors often reported being judged by others as healthy although they felt physically and emotionally vulnerable. This made it difficult for a number of survivors to explain their supportive others, such as partners, relatives, friends or work colleagues, how they felt and what they struggled with. Some partners added that the survivors they cared were thus often reluctant to talk to others about their feelings and well-being as they did not wish to have to explain their disease and symptoms. However, they needed to share their thoughts and feelings with someone. In most cases this was their partner who was already familiar with their situation and did not need further explanation of their diagnosis, symptoms or treatment.

“*From the outside you seem healthy, as the acute phase has been over for some time. And the people around you they don't really recognize and understand that there are the negative long-term effects (*=*of alloHSCT).” (female survivor, 44 years, two years post alloHSCT)*

“*Well, you did have the need to talk. I remember last Christmas when you really talked about all of this quite extensively (*=*your disease and the side-effects of treatment). Do you remember?” (female partner, 65 years)*

### Time Post Discharge as a Dynamic Process With Individual Peaks of Burden

Survivors' care experience after being discharged from hospital was often seen as a dynamic process where individual survivors experienced various peaks of burden at different stages of the care trajectory, i.e., times that were perceived as particularly challenging, both physically and emotionally. A significant part of participants reported that these peaks occurred at the time of cancer diagnosis, but also at discharge. This was because survivors felt that leaving the perceived safety provided by the hospital system and arriving back home after receiving alloHSCT fueled their fear of doing something wrong when managing their symptoms and care. Most survivors also experienced a great degree of burden and distress caused by the intermittent nature and exacerbation cGvHD and lack of response to therapy. Almost all survivors also suffered from having to isolate themselves to reduce the risk of infections. Not being able to leave their homes and being dependent on partners and other family members was seen as particularly burdensome.

“*On the day of discharge, I was at completely down. I was really devastated. My first reaction on the day of discharge, when my wife picked me up, was: If that [*=*the cancer] comes back, I won't go through this again.” (male survivor, 62 years, five years post alloHSCT)*

“*You don't get any support. You are discharged. Then you are at home. You get your pills and then the nurses and clinicians say: ‘If anything happens, please ring us and you can come in straight away, but that doesn't help you very much at home.”' (male survivor, 62 years, one and a half years post alloHSCT)*

“*I was so used to being among people, always in company. And then me being at home on my own […] that was the worst.” (female survivor, 47 years, four years post alloHSCT)*

Partners highlighted survivors' suffering when coming home, particularly due to their dependence on partners' care. They reported helping survivors through this difficult time, especially by providing support with coping emotionally with their disease and treatment, helping with practical problems, such as attending clinics, paying bills or keeping the household, and supporting survivors understand the information provided by their clinicians and becoming involved in decisions regarding their care.

“*I thought: How can we cope? Because he [*=*the survivor she cared for] practically had to be nursed when he came home. And I had a job to go to. One of us had to keep up a job.” (female partner, 54 years)*

“*I always thought we would overcome this. Of course, sometimes you struggle. But sometimes you feel joy. (…) We learned to live with the disease. Well, we always have to work on it and learn to be tolerant (…)” (female partner, 65 years)*

“*It's crazy what this does to you (*=*the alloHSCT). I just remember when he came home (*=*from hospital), he was done. He could hardly walk. I had to shower him, clean the whole house, wash the curtains, change the bed sheets every day. It was extreme.” (female partner, 49 years)*

Due to their reduced level of physical and cognitive function a significant part of survivors and their partners were forced to reduce their workload, work part-time or quit work completely. Younger survivors often had to take a break from university or vocational training resulting in a great degree distress and insecurity regarding the future. Apart from the financial struggle, having a job meant having a purpose in life for most survivors as they wished to perceive themselves as valuable members of the society. Most survivors who lost their job felt useless, socially isolated or bored.

“*We (*=*partner and survivor as independent entrepreneurs) have limited our working hours so that I can look after my wife.” (male partner, 63 years)*

“*I cannot go to work anymore. It is no longer possible. It is very hard for me to understand that.” (female survivor, 57 years, two and a half years post alloHSCT)*

“*My money has vanished altogether, about 1000 euros per month gone. My husband took six months off. Honestly, I have to say: all our savings were completely used up.” (female survivor, 47 years, four years post alloHSCT)*

“*Every time I went to the letter box I felt anxious because there were more bills […] I didn't want to make that experience, no one would.” (female partner, 65 years)*

A number of survivors suffered from having to take high doses of numerous different types of drugs in order to manage the side-effects of alloHSCT. They also struggled with the lengthy and frequent check-up visits at the clinic. Failed attempts to reduce medication and stagnation in rehabilitation were perceived to be particularly challenging. Some survivors reported that cGvHD and trying to manage its symptoms completely took over their lives, reducing their hope that things might improve and impacting their drive to make plans for the future.

“*[M]y systemic immune suppression has not changed. Every single tapering strategy has failed. I cannot reduce the high level of suppression. That has left me emotionally drained.” (female survivor, 44 years, two years post alloHSCT)*

“*[W]hen I take these tablets, 26 per day, when I take half of them in the morning, I feel so sick. I just want to go back to bed. Because all the energy that my body has left is used up in the process of metabolizing the drugs.” (female survivor, 51 years, one and a half years post alloHSCT)*

“*This GvHD determines my life. That's it. To some extent I have lost hope that I will ever be able to reduce all this medication. ” (female survivor, 57 years, two and a half years post alloHSCT)*

### Transparent Communication and Support With Patient Empowerment

Most survivors indicated that their treating clinicians had told them that receiving alloHSCT would be tough and time-consuming. However, they were nevertheless surprised and sometimes even shocked by the extent and intensity of the treatment side-effects and by how much they would affect their lives. A significant part of study participants only realized post-transplant that alloHSCT would not provide the quick cure they wished for, but that the recovery period would be a crucial and burdensome part of their care trajectory. Partners also highlighted the feeling of not being prepared for the impact the transplant had on the survivors they cared for, particularly the changes on personality, the changes related to their bodies and the cognitive difficulties they had to cope with post-transplant.

“*And I have to admit, I did not expect the treatment complications.” (female survivor, 57 years, two and a half years post alloHSCT)*

“*And that was a totally new experience to acknowledge or to learn to acknowledge that you are not cured but that the process of healing was continuing and that the time after the transplant was a very important time.” (male survivor, 62 years, two years post alloHSCT)*

“*From one day to the next I thought there is another man lying in the room. (…) His head and neck were swollen. I couldn't believe that it was him. It was a shock.” (female partner, 62 years)*

Survivors in this study varied greatly in terms of their preferences for the type and the amount of information they wished to receive. Some wanted their clinicians to provide all available details of their diagnosis, prognosis and treatment options, others preferred to only receive general information that helped them understand what happened to them. These survivors felt that too much information would scare them. All survivors appreciated if information was given to them in lay-language and in a structured manner to ensure survivors and their partners would not be overwhelmed by the amount of information they received.

“*As a layperson you cannot understand that jargon when you get confronted with it. There should be a training program for clinicians to help them to explain something in a way that the patient can understand.” (female partner, 65 years)*

“*You get so much information that you cannot really understand what has just happened. The first information came like a landslide.” (male survivor, 68years, two years post alloHSCT)*

“*It's a very personal thing [*=*information needs and preferences]. Everybody is different. Clearly there are things I need to know. But there are also things when I think, just leave them. I don't want to know. Yes, I would worry too much.” (female survivor, 68 years, two years post alloHSCT)*

Some survivors also highlighted the importance of their clinician being mindful about how they provide information and how this is received by survivors and partners. A number of survivors reported that clinicians thought out loud what they saw when examining them or reading their test results. Clinicians often said what they concluded from these examinations or test results without actually addressing survivors directly or filtering the information in order to make it understandable and less anxiety provoking for not medically trained people. Consequently, survivors immediately thought that something was wrong causing them a great degree of distress that could have been prevented by more careful means of communication.

“*The ultrasound image showed up something on my thyroid. And then they [*=*the treating clinician] say to you: ‘Well, we'll definitely have to investigate that. You were getting radiation therapy and some tumors can come back.' And my reaction was: danger, danger, danger!” (female survivor, 57 years, two and a half years post alloHSCT)*

Many survivors reported not adhering to their prescribed care, e.g., their medication or isolation, as they were overwhelmed by the myriad of instructions and limitations they had to integrate into their daily lives. Clarifying the reasons for these limitations, prioritizing some over others and providing practical recommendations for how to put the instructions into practice were perceived important aspects to help survivors increase adherence to their prescribed care. Survivors reported that liaison nurses provided a lot of support in this area, particularly by showing them how to administer medication, answering questions regarding how to reduce the risk of infection or giving advice on nutrition. These liaison nurses involved a group of specially trained nurses who provide tailored follow-up care. The liaison nurses also helped survivors by educating partners on the recommended care, as they were the ones who often implemented the provided guidelines, especially in the period immediately after discharge.

“*Maybe not just what you shouldn't do, but also an explanation why [*=*certain things should be done] would be good.” (female survivor, 51 years, two years post alloHSCT)*

“*I was looking for hours to find the right sunscreen and to check all the products, to understand the pros and cons and check the ingredients for those I should stay away from. Maybe you could improve that a bit by making sure you have a product to start with and then you can go and look for others to continue with.” (female survivor, 51 years, two years post alloHSCT)*

“*If we had known that you can eat pealed raw veggies, we would have had a carrot salad or something like that a long time ago. But we avoid all of this, just to be safe.” (female partner, 31 years)*

Some survivors wished for more information about alternative medicine and how it could be used to reduce drug-interactions. Others would have appreciated further details on the clinic they attended, including more comprehensive readily available and trustworthy information on the experience and qualifications of the clinicians who treated them. This was because most survivors felt that centers varied greatly by the quality of care that was provided, with some not being perceived to be up-to-date with current clinical guidelines and recommendations on optimal patient-centered communication and care prior, during and post alloHSCT. Some survivors wished for clear, publicly available information on each healthcare center, for instance regarding the number of transplants administered per year and clinicians' experiences with treating different hematological conditions.

“*I believe that many patients would like to use alternative ways or means in order to avoid using chemical drugs. And they could do with more support on that.” (female survivor, 51 years, two years post alloHSCT)*

“*I think it is a great problem to find a clinic that doesn't just do a sufficient number of transplants but also has experience with very different types of disease because that ensures that follow-up care will work well. […] But that information doesn't exist. The public relations department [*=*of each clinic] writes nice adverts on how great everything is and all the things they do at the clinic but quite often there is a lack of information on things like: How many allogenic HSCTs do they do? How many autologous per year? I don't know how to improve this but maybe you have to force clinics to communicate in a more transparent matter. Some people may attend the clinic closest to their homes although they won't receive the best care there.” (female survivor, 57 years, two and a half years post alloHSCT)*

Easy access to appropriate support with physical activity was seen as another facilitator for optimal survivorship care. Most survivors found it important to increase their physical fitness and felt this would help them cope emotionally with their new situation. They reported that physical activity allowed them to increase their self-confidence, optimism and self-effectiveness, and receive a welcomed distraction and timeout from daily routines and concerns. Despite this, most struggled with attending open fitness centers or sports classes, since they were concerned with the risk of infection and of potentially crowded exercise areas. Some also felt that open fitness centers may not be sufficiently tailored to their physical and mental well-being and may thus expose them to exhaustion. They wished for courses and fitness plans tailored specifically to their specific needs after alloHSCT and guided by a trained instructor. Survivors also indicated that such programs should be better communicated in order to raise awareness among survivors and treating clinicians.

“*Sport has always been good for me because doing sports also provides psychological support.” (female survivor, 49 years, two years post alloHSCT)*

“*When I got home from hospital, I had no energy. But I needed to get out and (walk) a little bit further every day. I could draw a lot of energy from that. I always say, I am walking away from my illness.” (female survivor,64 years, three years post alloHSCT)*

“*I cancelled my sports class because I didn't go there anymore. I can't do any exercises on the floor anymore due to my hip and back. And the floor is contaminated, and I don't want that either. Nothing is being done here in X [*=*city where the survivor lives] … I mean that they should try to offer sports classes that are suited for HSCT-survivors.” (female survivor, 57 years, two and a half years post alloHSCT)*

### Continuity of Care and Help With Claiming Social Benefits

Being discharged from hospital was particularly challenging for survivors who lived far away from the clinic they were treated at. In case of emergency, they often attended an emergency department close to their homes where they did not feel their disease and concerns were treated adequately. Some felt that this was because of smaller clinics' lack of experience with providing specialized care to alloHSCT-survivors. A number of partners reported not having received adequate care when they had to take their partner to the nearest emergency department.

“*And there is always the fear something could happen. And what makes it worse for us: we live 138 km away from X [*=*place of the clinic they attended]. […] What I found most devastating was that every time I had to take my husband to the emergency department […] the people there didn't take us seriously and sent us to the waiting area. And I had to get up and fight for my husband to be treated immediately. Those were the worst moments.” (female partner, 59 years)*

A number of participants reported a lack of communication between their treating specialists, general practitioners (GPs) and the emergency departments they attended. GPs and local emergency departments were often not aware of the special needs of alloHSCT-survivors resulting in survivors and their partners having to close the gap of communication in order to receive appropriate care. Survivors and partners in our study wished for better information exchange between all members of their care team, including primary, secondary and tertiary care clinicians. They suggested continuing education for GPs as well as better documentation and communication of the details of each survivor's care trajectory. Also, survivors noted the importance often having the same clinician looking after them during their follow-up check-ups. This allowed them to build a doctor-patient-relationship which is based on mutual trust and helped survivors gain confidence in their prescribed care. Such a doctor-patient-relationship was also seen as important condition for survivors feeling confident to talk about openly their individual problems and wishes.

“*Involving the patient's GP [*=*is important]. They should set aside an hour or two between the GP and the specialist. The GP is the extended arm [*=*of the specialist], aren't they? This would not cost a lot […] that they know exactly what they are doing.” (male partner, 48 years)*

“*Every time I build a relationship with a doctor they leave soon after. It is really annoying. Because you always talk a bit about yourself. First, the doctor is a stranger to you, then, during the second visit, you start to get to know them, and again during the third visit, and when you want to meet them for the fourth time they are gone.” (female survivor, 57 years, two and a half years post alloHSCT)*

Survivors and partners in our study wished for more tailored help with claiming social benefits and seeking further practical support. They suggested introducing a temporary care level for the times they struggled most post-transplant.^1^ Others reported an unmet need for improved access to paid housekeepers and additional sick leave for partners. A significant part of study participants suggested that clinics provide a dedicated social worker who is familiar with claiming social benefits and can thus support each individual survivor and their partner. They noted that it would be important to have such support during and post discharge as help with claiming social benefits often stopped once survivors left the hospital. As soon as they were back at home, they often felt left alone with having to communicate with different authorities and deal with various issues related to their health and pension insurances. Given that some clinics did not directly bill their insurers, a number of survivors found it particularly time-consuming and burdensome having to forward each and every treatment bill to their health insurer themselves.

“*It would be really good if there would be a temporary care level for the first six months, so that someone can drop in at your home once a day.” (female partner, 54 years)*

“*What really annoys me was the letter from the pension insurance. I think my blood pressure went up to 200 […] I had to get my hips done and then they said: ‘Well, you have two new hips, now you can work six hours a day and more.' And then I thought: Ok, how should I do that? I cannot stand for a long period of time, I cannot sit. So, it really doesn't work.” (female survivor, 57 years, two and a half years post alloHSCT)*

“*Starting from the pension insurance on to the health insurers, to the benefit agency, and so on. It was an odyssey, it really was. It is unnecessarily difficult. It should be made easier. And I really believe that the people who claim something really need it.” (female partner, 65 years)*

“*That [*=*applying for pension and to receive disability allowance] was a mental strain which I couldn't stand any longer in the end. Finally, I said to myself I don't care anymore. I don't want to go on anymore. I do what they say even if I have financial disadvantages. I don't have any strength anymore.” (female survivor, 64 years, three years post alloHSCT)*

“*Many hospitals don't deal with the health insurances directly, they simply don't want to. That is then left to us. I have to pass it on to the right people. […] I have put the money into a separate account, tens of thousands of euros which I transfer into the account and then I wait that the health insurer reimburses me for the bills.”(female survivor, 64 years, four years post alloHSCT)*

Survivors treated by liaison nurses reported that their team helped them through this difficult time by offering care and support with questions or concerns they had. The liaison nurses fostered continuous care by providing survivors and their partners with a list of healthcare providers they could contact if they needed help with social support or managing their care at home. Liaison nurses were also seen as a facilitator for multidisciplinary care involving primary, secondary and tertiary healthcare services and maintaining communication between different members of survivors healthcare team. Survivors and partners who received this type of care greatly appreciated the continuing and tailored support.

“*And there is this - and I really have to praise it - this wonderful system of building a bridge between hospital care and care at home. There is someone who takes the time to tell us what to look out for at home.” (male survivor, 62years, two years post alloHSCT)*

“*You always have the feeling that you can call the team of the liaison nurses if you need to.” (female survivor, 68 years, two and a half years post alloHSCT)*

“*You just feel well looked after within this system of liaison nurses.” (female survivor, 46 years, two years post alloHSCT)*

## Discussion

Our report builds on existing evidence (19, 36, 37) and highlights the experiences and needs of hematological cancer survivors including their partners following alloHSCT in a European context of care. Survivors commonly reported the negative impact of late effects and GVHD on their well-being across different stages of survivorship. Notably, survivors also provided new insight into the difficulties associated with accessing specialized care, being empowered to self-manage their condition and care as well as accessing social benefits they were entitled to. Our findings provide new insights into an understudied area of research and highlight the cornerstones of optimal survivorship care post alloHSCT.

Many survivors and partners see the survivorship trajectory as “dynamic process” with individual peaks of burden, a diversity of long-term treatment side-effects and a clear need for transparent communication (5, 18, 33). These peaks of burden did not only occur at diagnosis but also at discharge requiring intense psychosocial follow-up care involving (peer) support groups and patient's advocacy groups. Survivors felt that leaving the perceived safety provided by the hospital system and arriving back home after alloHSCT fueled their fear of doing something wrong when managing their symptoms and care at home. Many alloHSCT-survivors in our study struggled with the heightened risk of infection, fatigue, limited fitness and neurocognitive decline and were reluctant to share their thoughts and concerns with others than their partners as reported by previous studies (14, 48, 49). They did not feel adequately prepared for this transition from acute to chronic disease and struggled with the disruption this caused in their personal life plans and goals. They reported that peaks of burden occurred during exacerbation cGvHD and lack of response to therapy. This is in line with a previous study of Jim et al. describing the intermittent nature of cGvHD as a source of burden and distress and highlighting the potential benefit of patient education materials to increase knowledge and self-efficacy in dealing with side-effects of alloHSCT (50). It may be helpful to provide further education of survivors and support persons about the side-effects of alloHSCT prior to transplant and deliver guidance on how to identify and communicate their concerns and wishes, so that they can be addressed in a timely manner.

A model of care found particularly helpful by our study participants is the system of liaison nurse, provided in some of the clinics participants attended. This system involves specialized nurses providing individually tailored advice on alloHSCT-survivors' supportive care post discharge, including constant low threshold counseling on survivorship issues during the recovery process post alloHSCT. A number of studies suggest that involving liaison nurses can improve the continuity of care and patient outcomes (51, 52). One of the key elements of involving liaison nurses is that counseling already starts before alloHSCT to help build a solid and continuing relationship before further medical interventions are required. The need for such models of care to help manage alloHSCT-survivors cope with their symptoms and care has been highlighted previously (36, 53). However, more intervention studies are required to examine the effects of such models of care in alloHSCT-survivors and their partners. Future research should also explore which elements of tailored care planning work best in different settings and examine barriers and facilitators to their implementation into routine care.

Taken together, our results indicate that care for alloHSCT-survivors does not allow a “one-fits-all”-strategy but requires healthcare service delivery that is tailored to the individual needs and circumstances of each survivor. Tailored care post alloHSCT has been shown to increase survivors' capacity to self-manage their care and even avoid loss to follow-up (54). McConnell et al. examined different subgroups of cancer survivors highlighting that tailored care planning is a key element in increasing survival, treatment success and patient satisfaction levels (55). Wagner et al. also emphasized the importance of tailored care planning to support survivors' self-management and help them and their supportive others cope with chronic illness (56). Tailored care planning can further enhance health-related quality of life, reduce depression and reduce healthcare costs related to visits to emergency departments (57, 58). It has also been suggested that compared to usual care individualized care plans can lower distress scores and improve the mental domain of QoL among HSCT-survivors 1–5 years post-transplant (59). Despite this, the use of tailored care planning is still relatively low in European countries, with most studies conducted in the areas of chronic obstructive pulmonary disease (COPD) and diabetes patients (60, 61).

Participants in our study also indicated the need for continuity of care after leaving the hospital. As also shown by others, we noted that frequent changes of treating clinicians was seen problematic as patients struggled with building trust in their clinician and create profound relationships (62). They wished for better interdisciplinary documentation and communication, for instance through multidisciplinary teams. Another way of improving collaboration between clinicians from different disciplines and healthcare services might be the use of electronic tools, such as electronic health records. A number of studies in other settings suggest that this can improve quality, safety and continuity of care as well as interprofessional communication through quicker and easier access to medical data allowing more efficient and patient-centered care (63–65). Better interdisciplinary documentation and communication may be particularly important when trying to improve care for alloHSCT-survivors provided by local emergency departments and GPs, who are often not familiar with the special needs of alloHSCT-survivors and the requirements for optimal care post-transplant.

Support in gaining social benefits was seen as further facilitator for optimal alloHSCT-survivorship care given that many survivors did not know what they were entitled to and often felt overwhelmed and frustrated when trying to obtain social benefits. Our data suggest challenges in claiming social security benefits for orphan diseases, such as cGvHD, caused by numerous problems. These include difficulties with understanding the benefits survivors are entitled to, finding the responsible authority and contact person and submitting correct applications. Participants need professionals familiar with legal provisions and practical aspects of social support to provide them with timely information, e.g., prior to the transplant. This could involve patient navigators who help survivors with taking-up or returning to employment or complete their education (66). Such navigators would be familiar with the situation of each survivor and coordinate their healthcare support. They could help engage survivors in proactive roles in their care management, facilitate transition from hospital to community care and improve communication between the members of their treatment team (66). Employing patient navigators to help alloHSCT-survivors and their informal caregivers access care and further social support mechanisms could also improve adherence to their prescribed care (66, 67). A recent study of Berezowska et al. found that patient navigation can increase satisfaction with care among survivors and healthcare professionals, improve survivors' self-efficiency and reduce distress (68). In some settings, bridging clinics already take on some of these tasks and help patients claim social benefits. However, there may be a need support liaison nurses by providing patient navigators who are trained more specifically for this purpose. Digital solutions could also be used to support liaison nurses. For instance, Maher et al. developed a HSCT-specific health IT tool called *BMT-Roadmap*, that promotes survivor education and involvement during hospitalization (69). A *BMT Roadmap 2* for the outpatient setting is currently being considered (70) and could also be used to help survivors claim social benefits. Further attempts to use eHealth to improve care post-transplant highlight the enormous potential of eHealth in promoting interdisciplinary collaboration and patients' satisfaction (71, 72). However, more research is needed to validate such interventions (68).

Our data further suggest that survivors' emotional and mental well-being, their satisfaction with and adherence to care could be improved by specific and continuing training for clinicians on patient-centered communication, for instance on more careful communication on how to deliver test results. Survivors in our study reported that clinicians often delivered test results accompanied by their first thoughts on the interpretation and potential implications of these results. This often caused survivors to assume the “worst case” scenario resulting in substantial anxiety and distress (73, 74). Participants also mentioned the importance of understanding the reasons for their prescribed care post-transplant, including detailed information on their medication and infection prevention measures. Survivors highlighted that this would help increase their feeling of being actively involved in their care and increase their adherence to their prescribed care. This is in line with several studies in other chronic settings which suggesting that survivors are more likely to be able to self-manage and adhere to their care instructions if they are empowered to do so by being told why specific care instructions were given and what important role they play in their own recovery and survivorship care (75–77). Tailored communication trainings could also help clinicians enable and empower their patients and informal caregivers (78). This may be particularly important if patients or their support persons as informal caregivers feel they have misunderstood certain aspects of their diagnosis, prognosis or care but do not dare to mention this. Training in patient-centered communication could help assist patients and informal caregivers with expressing these unmet information needs, so that clinicians can address them.

Participants in our study also indicated that communication could also be improved by providing publicly available information on center characteristics such as the number of transplants administered per year, the number of different diseases types treated and how survivorship care is being delivered at each clinic. Similar efforts have been made in other areas of medicine suggesting that such information allows survivors to make informed decisions regarding their care (79). This has been show to improve patient trust in their clinicians and may also increase healthcare providers' commitment to improve the quality of care (80, 81).

A number of outcomes, such as adherence to the prescribed care, could also be increased by involving the triad of clinician, survivor and their informal caregiver (82). A number of studies suggested that involving survivors' supportive network is a key element of optimal patient-centered care (83, 84). Our data confirmed this as partners played an important role in helping survivors cope with the long-term effects of alloHSCT. Partners and other members of survivors' supportive network often provide one of the most important sources of information and advice for survivors, and can play an important role in treatment decision making (22). They have been found to help improve a number of survivors' outcomes, including mental health and long-term survival (85–88). Informal caregivers can further provide valuable additional information on aspects of care survivors struggle with and how to overcome them (86). More research is required that involves the views of survivors' supportive others on how to improve their care (21).

Finally, many survivors mentioned physical activity as an asset to cope with the long-term effects of alloHSCT, increase their self-confidence, optimism and self-effectiveness, and as a welcomed distraction and timeout from daily routines and concerns. This is in line with previous studies suggesting that physical activity can improve survivors' outcomes, such as decreased hospitalization and better physical health (89, 90). Wiskemann et al. found a positive correlation between non-relapse mortality and physical activity (91). However, many survivors in or study struggled with attending general fitness classes given a perceived risk of infection. In light of these findings and given the current COVID-19 pandemic online exercise classes may help provide survivors with an opportunity to stay physically active. However, further research is required that investigates how to design and implement physical activity classes that are tailored to the special needs and circumstances of alloHSCT-survivors.

## Limitations

Our results do not intend to be numerically representative. We employed theoretical sampling which is typically used in qualitative work and aligns with the aims of this study to provide fresh in-depth insights into survivors' needs and experiences, which is a recognized component of optimal care (92). Our data also make suggestions for how to improve care in this area which can guide clinical practice and resource allocation. However, due to differences in healthcare services and systems our findings may not be generalizable to other settings. Our findings highlight that partners may provide valuable information on survivors' needs and experiences by complementing their narratives and raising topics not initially raised by survivors. Participating survivors did not feel intimated by the presence of their partners but appreciated sharing their thoughts and experiences with the support of their partners. This is in line with previous studies highlighting that interviewing individuals and their partners together could generate richer data than interviewing them separately due to the interaction between the interviewees leading to a more holistic presentation of the studied phenomena (93–95). However, it was not possible to include partners for all of our interviewees due to difficulties with recruitment, mostly due to a lack of time. Theoretical sample ensured the representativeness of the included participants in terms of age, gender, and socioeconomic status. Despite this, a significant percentage of survivors were married and attained year 10 or lower of education which could potentially limit the generalizability of the results. Also, we involved a limited number of centers and participants received alloHSCT at different centers and thus received different transplant-related education, and different support once discharged. This may affect the unmet needs found. Finally, there may have been recall bias since participants were asked to comment on HSCT which was a number of years ago for some.

## Conclusion

This qualitative study provides novel insights into alloHSCT-survivors' experiences and needs post-transplant. It also includes partners' views with the aim to add further information on survivors' needs and how care in this area could be improved. Our results suggest that the time post discharge is particularly challenging for most survivors who also often struggle with neurocognitive decline and the need to reduce their workload or quit work altogether during long-term survivorship. Survivors in our study wished for further efforts to ensure patient-centered, multidisciplinary, holistic, continuous survivorship care that is tailored to their specific needs. Additional support with navigating the healthcare and social service system as well as help with understanding and participating in their care could increase adherence to care, self-effectiveness and physical and mental well-being. This would involve improvement in communication and education along the whole transplant continuum, so survivors can understand what they are dealing with and what resources they may be entitled. This may be achieved by having specialized patient navigators and liaison nurses. Finally, intervention studies are required to test the effectiveness of the suggested strategies and, if found to be effective, investigate how these strategies could be best implemented into routine cancer care.

## Data Availability Statement

The raw data supporting the conclusions of this article will be made available by the authors, without undue reservation.

## Ethics Statement

The studies involving human participants were reviewed and approved by ethics committee of the University of Regensburg. The patients/participants provided their written informed consent to participate in this study.

## Author Contributions

MP: conducted the interviews and wrote the manuscript. JL: supervised the project. EH: supervised the project and commented on the manuscript. AB: added to the discussion of the data. DWe: recruited patients. ME: edited the manuscript. DWo: initiated the project and supervised the project. AH: initiated the project, performed the interviews, and wrote the manuscript. HS: contributed to the discussion and manuscript. All authors contributed to the article and approved the submitted version.

## Conflict of Interest

HS has received financial compensation for advisory boards (Incyte, Janssen, and Novartis), speaker's fees [Novartis, Incyte, Jazz Pharmaceuticals, the Belgian Hematological Society (BHS) and Takeda], travel grants (AbbVie, Celgene, CIBMTR, EBMT, Gilead, and Incyte), and research funding (Novartis and the BHS). She also frequently served as a volunteer for the BHS, the CIBMTR, EBMT, and EUPATI. DWo received honoraria from Novartis, Behring, Mallinckrodt, MACO, and Incyte. The remaining authors declare that the research was conducted in the absence of any commercial or financial relationships that could be construed as a potential conflict of interest.

## Footnotes

^1^In Germany, the benefits of each person's long-term health insurance cover are provided in different stages which are also referred to as “levels of care.” These levels of care are based on the severity of each person's condition and help categorize and classify the health insurance claims made.
