# Supplementary material for: “This Graft-vs.-Host Disease Determines My Life. That's It.”—A Qualitative Analysis of the Experiences and Needs of Allogenic Hematopoietic Stem Cells Transplantation Survivors in Germany
Source: Front Public Health. 2021 Jul 1;9:687675. doi: 10.3389/fpubh.2021.687675 (PMC8280766; doi:10.3389/fpubh.2021.687675)
Supplement: Supplementary file 1 [file Data_Sheet_1.docx]

# Appendix

**Appendix 1: Interview guide**

**Opening script detail**

Hello, my name is XY. I am working at the University Hospital Regensburg.

- (If required: We recently contacted you to arrange a time for an interview.

Are you still happy to participate?

1. *If yes, proceed with the following questions.*
2. *If no, ask why and whether to postpone interview. If patient does not want to be contacted again, thank for time, offer to be contacted again if they change their mind and end interview.*

Is this time still suitable for you?

1. *If yes, proceed with the following questions.*
2. *If no, arrange a suitable time for an interview.*)

- I would like to talk to you about your life after the stem cell transplant.
- This will take up about 45 minutes of your time.
- Before we begin, I would like to ask you whether you are happy with the fact that our conversation will be recorded and typed. This is just to ensure that we can analyze the data appropriately. Of course, everything you say will remain confidential. The results of this study cannot be linked to your identity.

1. *If yes, proceed with the following questions.*
2. *If no, explain that all data will be de-identified and ask for permission to record the interview. If patient still denies, ask for allowance to take notes during the interview.*

- We can stop the interview anytime or skip questions you don’t feel comfortable answering. Please just let me know when you feel you would like to stop or skip questions.
- Also, I would be grateful if you could sign the consent form and the sheet about data protection. I am happy to give you a copy of the signed documents if you would like.

**Preamble**

- We know that people’s lives may not be the same before and after a stem cell transplant. Of course, doctors focus on cure but surviving cancer may also mean that a patient struggles with the long-term consequences of their treatment.
- This research aims to explore this. The results will inform policy and clinical practice.
- Now I would like to talk to you about your life after the transplant. I am interested in how the transplant affected your life. I am also interested in how you, as a partner, perceived this.
- First, it would be great if you could tell me everything that comes into your mind when thinking about the impacts the transplant had on your life. Also, I would be grateful if you (=the partner) just add your views and experiences to this.
- You can take as much time as you wish. I will just listen carefully, take a few notes and ask further questions later on.
- Now, could you tell me: How did the transplant and its side-effects impact on your life?

**Additional topic areas (Partners were asked to add to survivors’ narratives, e.g., by prompting them to explain how they experienced certain aspects the person they cared for had raised and whether they would like to add to what the survivors has said)**

How did the transplant (and the short- and long-term effects of it) impact on your:

- Physical wellbeing?
  - Have you had any pain, nausea/vomiting, fatigue?
- Emotional wellbeing? Have you felt any:
  - Anger, despair, fear or hopelessness
- Did you need any further information, e.g. on:
  - Your diseases, treatment, symptoms, available support services etc.?
- Has the transplant affected your daily routine and practical aspects of your life? (e.g. household, grocery shopping)
  - How has the transplant impacted on your work life?
- Did you have any further practical needs, e.g.:
  - Finances (e.g., time off work, treatment costs, being able to pay rent/loans)?
  - Child care?
  - Housekeeping?
  - Legal issues?
- Have the side-effects impacted on:
  - How you see yourself? (e.g. self-confidence, self worth)
  - Your body image?
  - Your coping?
- How did the transplant (and its side-effects) impact on your thoughts about the future?
  - Has the transplant changed your plans for the future? If so, in what way?
- Did the transplant affect your thoughts about:
  - Life in general
  - Suffering
  - Pain?
- Has the transplant affected your relationship to others?
  - Partner, family, friends
  - School, work
- Was it difficult to meet family and other social obligations? (e.g. looking after children or other family members you care for)
- Did it influence the desire to go out and socialize again?
- The next questions may be a bit sensitive, so you don’t need to answer them if you don’t feel comfortable with it: How did the transplant impact on your sexuality or intimacy?
  - Feeling of being a man/woman?
- Do you think things would be very different if you hadn’t had the transplant? If so, in what way?
- Which support did you have in dealing with the consequences of the transplant?
  - How do you feel about this support?
  - What other support could have been offered?
- If you were politician, what would you change regarding the care for people who had a stem cell transplant? In other words: If we were living in an ideal world, what support should be provided to patients after the transplant?

**General questions on impacts of the transplant**

- What would you say, which of these things affected your life most?
- How did the impacts of the transplant change over time? (e.g. worsened when transitioning from hospital to home)
- After the transplant, have there been any particular events or complications that changed your life considerably?
- How do you manage the side-effects of the transplant? (e.g. medication, hospital visits)
  - How difficult is it to manage these effects?
  - Do you feel well-equipped to manage the effects of the transplant?
  - Have the effects of the transplant impacted on your relationship with the hospital staff? If so, in what way?

**Thank you.**

**Sociodemographics (collected from survivors and partners)**

Before we end the interview, I would like to ask you a few more questions about you and your background.

- Are you a German citizen? If not, please indicate what country you are a citizen of:
- What is the highest level of education you have completed? (e.g. Year 10/School Certificate or lower, Higher School Certificate, Trade or vocational training (e.g. TAFE or college), Bachelor degree, Postgraduate degree, Other)
- Do you currently have a usual occupation? If so, what is your usual occupation?
- What is your employment type (e.g. full-time, part-time, and casual)?
- What is your current marital status? (e.g. single or never married, divorced or widowed, living with a partner, married)
  - Are you living with your partner?
  - Do you have children? Younger or adult?
- (For partners as this information could not be retrieved from survivors’ medical records: What is your year of birth?)

**Other comments**

- Do you have any other comments that you would like to make? Have you got any questions?
